# Size-structured risk assessments govern *Daphnia* migration

**DOI:** 10.1098/rspb.2008.1088

**Published:** 2008-09-23

**Authors:** Lars-Anders Hansson, Samuel Hylander

**Affiliations:** Institute of Ecology/LimnologyEcology Building, Lund University, 223 62 Lund, Sweden

**Keywords:** migration, zooplankton, risk, ultraviolet radiation, predation, *Daphnia*

## Abstract

One of the more fascinating phenomena in nature is animal mass migrations and in oceans and freshwaters, diel variations in depth distribution of zooplankton are a phenomenon that has intrigued scientists for more than a century. In our study, we show that zooplankton are able to assess the threat level of ultraviolet radiation and adjust their depth distribution to this level at a very fine tuned scale. Moreover, predation risk induces a size-structured depth separation, such that small individuals, which we show are less vulnerable to predation than larger, make a risk assessment and continue feeding in surface waters during day, offering a competitive release from down-migrating larger animals. Hence, we mechanistically show that such simple organisms as invertebrate zooplankton are able to make individual, size-specific decisions regarding how to compromise between threats from both predators and UV radiation, and adjust their diel migratory patterns accordingly.

## 1. Introduction

Large scale animal migrations have fascinated naturalists and scientists for centuries, may it be seasonal bird migrations, dense flocks of savannah animals or aquatic migrations, such as the trans-oceanic migrations of eel (*Anguilla anguilla*), or mass migrations of cypriniol fish (Hansson *et al*. 2007; Bro¨nmark *et al*. 2008). Large scale migrations are often temporally repeated patterns triggered by, for example, harsh abiotic conditions, such as low temperature, predation or competition for resources. In freshwater ecosystems, as well as in oceans, many invertebrate zooplankton perform strong diel vertical migrations (DVM), which are generally directed from surface waters during daytime and back again during night (Stich & Lampert 1981; Hays *et al*. 1995). Although zooplankton are small animals, they are indeed numerous and these DVM are probably among the largest animal movements on the planet with respect to biomass (Hays *et al*. 1995).

In freshwaters, the herbivorous genus *Daphnia* is a key organism with considerable impact on food web dynamics and ecosystem functioning (Hansson *et al*. 2004). *Daphnia* has a positive phototactic behaviour towards visible light (Storz & Paul 1998), i.e. it will, if not threatened by UV radiation or predation, remain in surface waters where algal food is generally most abundant. Owing to its size and relatively inefficient protection against visually feeding fish, *Daphnia* is vulnerable to predation and is often absent or rare in waters with dense fish populations (Brooks & Dodson 1965; Hansson 1992). Although diel migrations may be affected by a multitude of processes (Ringelberg 1999; Lampert *et al*. 2003; Winder *et al*. 2004), by far the most common explanation is that they are a predator avoidance strategy, such that spending the light hours in deep, dark waters reduces the encounter probability by visually hunting predators, e.g. fishes (Zaret & Suffern 1976; Stich & Lampert 1981; Ringelberg 1991; Siebeck & Böhm 1994). This also implies that smaller species or individuals may show a less strong behavioural response to predation from large, vertebrate predators, such as fishes, than larger ones (De Meester *et al*. 1995; Winder *et al*. 2004). There are, however, examples of migratory behaviour in systems without fish predators (Williamson *et al*. 2001) and an alternative, more physiological, explanation is that diel mass migrations among zooplankton are avoidance strategy to ultraviolet (UV) radiation in surface waters during daytime (Hessen 1994; Leech & Williamson 2001; Rhode *et al*. 2001). Although there is support for both explanations, surprisingly few studies have addressed the UV and predation avoidance theories simultaneously (Ringelberg 1999; Hansson 2000; Leech & Williamson 2001; Hansson *et al*. 2007), and the consensus regarding when, where and how much each of the threats, UV and predation, affects the vertical distribution is still remote. The lack of knowledge and consensus is surprising since diel mass migrations of zooplankton is, indeed, a large scale and general phenomenon with far reaching effects on other trophic levels and on ecosystem functioning of both oceans and freshwaters. Moreover, the potential connection to global change, such as altered UV radiation, but also to invasive predators, makes it of crucial importance to understand causes and consequences of variations in vertical migration. In order to pinpoint the mechanisms behind this migratory behaviour, and disentangle the possible interactions between UV radiation and predation, we performed a large scale, long-term experiment where we manipulated both predation and UV risks. Hence, we mechanistically show that zooplankton are able to make individual, size-specific decisions regarding how to compromise between lethal, simultaneously occurring, threats from predators and UV radiation, and adjust their diel migratory patterns accordingly.

## 2. Material and methods

An outdoor mesocosm experiment was performed in Lund, southern Sweden (55.67° N, 13.5° E). Sixteen cylinders (diameter 0.37 m, height 1.0 m) were placed in two rows. Each cylinder was filled with 80 litres of tap water and water from a nearby lake (Dalby stenbrott) to a total volume of 107 litres. To the natural mixture of zooplankton from the lake we added *Daphnia longispina*, originating from a nearby pond, at a concentration of approximately 0.93 l^−1^. Food was provided once a week to each cylinder by adding 1 l of a mixture of *Scenedesmus* spp. and *Chlamydomonas* spp. at original concentrations of approximately 120 000 and 3000 cells ml^−1^, respectively. Our experimental design crossed UV radiation and fish predation risk in four treatments, randomly assigned to cylinders and replicated four times: visible light (V); visible light and fish cue (VF); the whole solar spectrum (UV); and the whole solar spectrum plus fish cue (UVF). Many prey organisms, including zooplankton, are known to react to chemicals excreted by predators (Brönmark & Hansson 2000). In our study, predator threat was mimicked by keeping a roach (*Rutilus rutilus*, 0+ or 1+) in a net cage (length 0.2×01×0.1 m) in surface waters, i.e. there was no actual predation on the zooplankton in the cylinders. The fish was exchanged once a week. In addition to UV from sunlight, extra UV radiation was supplied during 16 hours per day with one fluorescent lamp (Philips, CLEO Performance, 80 W, maximum intensity 350 nm) placed above each cylinder. The UV radiation reaching the water surface was controlled by different types of Plexiglas (Hansson *et al*. 2007), which either let both visible and UV radiation through (Röhm GS 2458; UV and UVF treatments) or cut out most of the UV radiation (Röhm GS 233; V and VF treatments). The cut-off is steep and almost complete at wavelengths below approximately 360 nm (Hansson *et al*. 2007). Overall the lamps caused an average increase of 37 per cent in daily integrated UVA radiation compared with a clear day in southern Sweden in the UV and UVF treatments, whereas the Plexiglas (GS 233) caused a 73 per cent reduction in UV in the V and VF treatments. Incoming radiation integrated over 24 hours (*I*_0_) was measured with UV sensors SUL 033 and SUL 240, connected to a logging meter IL 1400A (International Light, Newburyport, Massachusetts, USA). To estimate penetration of UV through the water, absorbance at 320 nm (*A*_320_) was regularly measured from water samples (Beckman DU 800 spectrophotometer). This wavelength is well correlated with the attenuation of UV radiation through water (Laurion *et al*. 2000). From *A*_320_, the diffuse attenuation coefficient at 320 nm (*K*_320_) was calculated (Kirk 1994; Morris *et al*. 1995). The daily integrated UV threat at 0.1 m water depth (*I*_0.1_) was then estimated for each cylinder and sampling occasion from *I*_0.1_=*I*_0_e^(−K320×0.1)^ (Hansson 2004). In order to keep periphyton production down the walls of the cylinders were swept with a brush once a week. Sampling was performed once a month between 10 May and 17 October 2006.

Vertical migration was monitored throughout the experiment by collecting samples from the surface (depth 0.1 m) and the bottom (depth 0.9 m) of the cylinders. At each level, 4 l of water were sampled for determination of *Daphnia* depth distribution at noon through tubing (diameter 9 mm) in the walls of each cylinder.

The 4 l sample from each cylinder and depth was concentrated through a 50 μm net and preserved with Lugol's solution. All zooplankters in each sample were counted using a multidish with eight sub-chambers, each 26×33 mm (Nalge Nunc, USA) and a dissecting microscope. In each surface and bottom sample, fifteen, or as many as could be found, *Daphnia* were measured from eye to the end of carapace in order to get a size distribution of animals at different depths. In May and October no size determinations were made since too few *Daphnia* were present. Animals were divided into size classes of approximately 0.2 mm from 0.5 to larger than 1.8 mm. The frequency, i.e. the number of animals in each size class, was then calculated and averaged over the season for each treatment and size class.

In order to test if fish from the population we used in the long-term experiment perform size selective predation, we assessed the actual predation pressure on each size class of *Daphnia* in an experiment using five aquaria (330×175×180 mm). The study was performed in a walk in incubator at a temperature of 17°C and a light intensity at the water surface of 1.1 μmol m^−2^ s^−1^. In each aquarium two roach (size 46.2±4.6 mm; mean±s.d.) caught in Lake Krankesjön, southern Sweden, were allowed to acclimatize (without food) for 24 hours. Prior to the experiment zooplankton (*Daphnia magna*) were cultured and just before the start of the experiment divided into eight identical portions with a plankton sample divider. *Daphnia magna* was used here since this species has a higher reproductive capacity than *D. longispina* allowing more thorough replication of the experiment. Three of the aquaria were randomly chosen as start samples and immediately preserved in Lugol's solution. The other five were put in each aquarium with fish, which were allowed to feed for 60 min. The water from each aquarium was then filtered through a 55 μm net and the zooplankton samples preserved in Lugol's solution. All zooplankton were measured at 20× magnification with an Olympus SZ 40 microscope and separated into size classes.

## 3. Results and discussion

**Figure 1:**
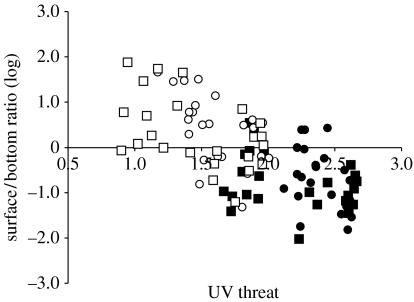
Vertical day-time distribution in response to UV threat. Abundances (log transformed) at surface and bottom expressed as a ratio (surface/bottom) along the experimental gradient of UV threat for *Daphnia longispina* (*r*=0.67; *t*_93_=7.78; *p*<0.001). Since the figure contains data from all samplings, it also illustrates the within treatment temporal variation in vertical position of the animals, reflecting the instantaneous UV threat. Treatments are: visible light only (V, open circles), visible light and predator cue (caged fishes; VF; open squares), ultraviolet radiation (UV; filled circles) and UV plus predator cue (UVF; filled squares).

The depth distribution of *D. longispina* during day, expressed as an abundance ratio between surface and bottom waters, showed a strong negative correlation with UV threat (*r*=0.67; figure 1). At the lowest UV threat (visible light only, with and without fish cue, V and VF treatments, respectively), *Daphnia* generally showed a positive surface:bottom ratio, i.e. most individuals were close to the surface. At higher UV threats (UV and UVF treatments; visible+UV radiation, with and without fish cue, respectively), most individuals chose to stay in bottom waters during day. However, the UV threat varied temporally between sampling dates due to variation in algal and DOC concentrations within each cylinder, i.e. also within treatments; a variation that was well mirrored in mean population depth distribution of *Daphnia*. This suggests that *Daphnia* are indeed able to, at very fine tuned temporal and spatial scales, assess the UV threat and adjust their depth distribution accordingly (Rhode *et al*. 2001). That the compound eye of *Daphnia* has a multichromatic photoreceptor system including UV sensitivity is well known (Smith & Macagno 1990), and also that they use this system to initiate negative phototaxis as a response to UV radiation (Storz & Paul 1998). Predation threat, or a combination of predation and UV (UVF treatment), did not lead to any further adjustments in the mean depth distribution. Instead, the predation treatments (VF and UVF) fit well into the regression slope of near −1 (*y*=−1.23*x*+2.16; figure 1), suggesting that UV was the major force behind the depth distribution of the population. This notion is further strengthened by a two-way ANOVA showing a strong overall effect of UV (*F*_1,94_)=58.34; *p*<0.001), but no effect of fish or interactions between fish and UV.

**Figure 2:**
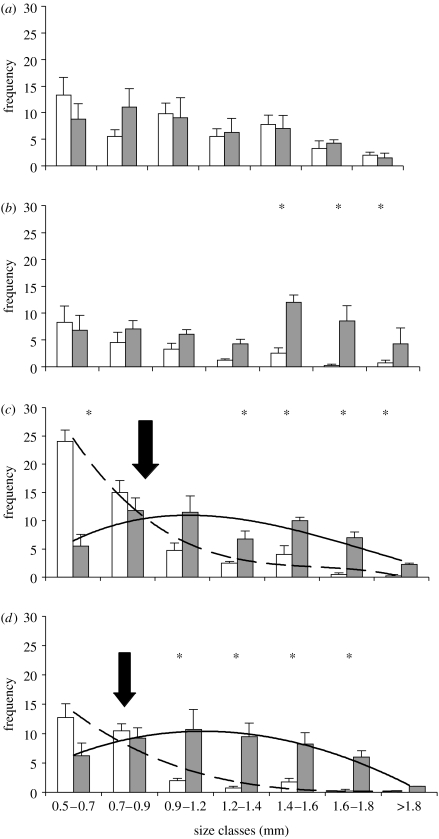
Predator induced day-time vertical distribution among size classes. Mean (±1 s.e.) frequency (number of *D. longispina*) in each size class (0.5 to greater than 1.8 mm) at surface (open bars) and bottom (grey bars) waters from June to September. Experimental treatments are: (*a*) visible light only (V), (*b*) ultraviolet radiation (UV), (*c*) visible light and predator cue (VF) and (*d*) UV plus predator cue (UVF). For clarity, best-fitted curve models are shown for surface (VF; dotted line; *y*=−0.24*x*^3^+3.85*x*^2^+21.38*x*+42.36; *r*^2^=0.97) and bottom frequencies (VF; solid line; *y*=0.08*x*^3^−1.63*x*^2^+7.70*x*+0.25; *r*^2^=0.70). Similar equations for UVF are: *y*=−0.06*x*^3^+1.34*x*^2^−9.38*x*+21.75; *r*^2^=0.92; and *y*=0.014*x*^3^−0.87x^2^+5.28*x*+1.93; *r*^2^=0.99). Stars show significant (*p*<0.05; MANOVA; general linear model) differences between frequencies of each size class in surface and bottom waters. Arrows indicate where frequency curves cross, i.e. at which size the majority of *Daphnia* chose to avoid surface waters.

Although our results clearly show that the UV threat is important in explaining the overall depth distribution of the animals, there were also differences in depth distribution among size classes of *Daphnia*. In the absence of any threat (V), no depth related size-structured distribution was recorded, but all size classes were evenly distributed among depths (figure 2). This was also the case in the UV treatment up to size classes of approximately 1.2 mm where larger size classes became more abundant in bottom than in surface waters (figure 2). However, in the presence of predator cues, small size classes showed strong preferences for surface waters, whereas larger size classes tended to prefer darker bottom waters (VF and UVF treatments; figure 2). In surface waters of both fish treatments (VF and UVF), the frequencies showed declines with increasing size of *Daphnia* (figure 2; dotted lines), whereas in bottom waters frequencies showed bell shaped responses with increasing animal size (figure 2). Hence, in the presence of fish, small size classes were over-represented in surface waters, whereas larger size classes were more common than smaller deeper down (figure 2). At the size where the models for surface and bottom frequencies cross (0.7–0.9 mm), the depth distribution was similar among animals (indicated with arrows in figure 2). In our study, the curves crossed at a size of approximately 0.9 mm suggesting that at larger sizes than this a majority of the *Daphnia* preferred bottom waters in the presence of predators (figure 2). Hence, in a situation with predators and visible light only (VF), small size classes took full advantage of the surface waters despite the predator threat (figure 2; MANOVA; *F*_1,6_=40.66; *p*<0.001). There was also a similar tendency, albeit not significant (figure 2; MANOVA; *F*_1,6_=4.24; *p*<0.085) in the UVF treatment. This suggests that smaller, younger individuals may be less responsive to UV, or that they are less sensitive due to transfer of photoprotective pigments from the mother (Green 1957; Siebeck *et al*. 1994). However, the difference between surface and bottom waters for the smallest size fraction was less pronounced than in the absence of UV, i.e. in the VF treatment (figure 2), suggesting that UV is to some extent experienced as a threat also for small size classes, whereas their response to the predation threat was negligible.

**Figure 3:**
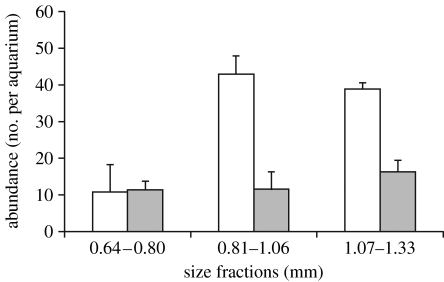
Predation pressure on different size classes of zooplankton. *Daphnia* of different size classes (0.64–1.86 mm) remaining after fish predation during 60 min. White bars show abundances of each size class before the experiment and grey bars after 1 hour of predation from fish.

Likely explanations of the dominance of small size classes of *Daphnia* in surface waters in the presence of predator cues is that there is less competition for algal food in surface waters as larger individuals migrate downwards. Moreover, small individuals are less vulnerable to predation than larger sized animals, allowing them to use surface waters during daytime (Johnsen & Jacobsen 1987). Accordingly, we found no predation on *Daphnia* at the size class below 0.80 mm in our predation experiment (*t*_6_<0.096; *p*>0.90; *t*-test), whereas all size classes larger than 0.81 mm suffered from a reduction of between 31 and 74 per cent due to predation (*t*_6_>2.60; *p*<0.05; figure 3). Hence, the fish predator indeed selected against the smallest prey, and this, together with the results in figure 2, suggests that *Daphnia* smaller than approximately 0.9 mm may not be expected to react to fish predator cues as strongly as large size classes since they are less preferred prey. Accordingly, when the overall mean size of *Daphnia* was only 0.83±0.13 mm (mean±1 s.d.) in June, there were no differences in size between treatments with and without predators either at the surface or at the bottom (table 1). In July, August and September, when overall mean sizes had increased to 1.05, 1.18 and 1.10 mm, respectively, the mean size of *Daphnia* in surface waters was generally below 0.80 mm in the presence of predators, and differences in size between surface and bottom exceeded 0.4 mm, corresponding to 35 per cent in length (table 1). This suggests that predator cues force larger individuals to seek refuge in bottom waters during daytime, whereas smaller individuals remain feeding in surface waters despite the perceived predation risk; a mechanistic explanation that has support from field observations (Johnsen & Jacobsen 1987).

Beside predation and UV radiation other factors may affect vertical distribution, such as temperature and food availability (Johnsen & Jacobsen 1987; Loose & Dawidowicz 1994). However, neither temperature, nor food availability, expressed as chlorophyll *a* concentration, differed among treatments in our experiment (RM MANOVA *F*_3,11_=0.13, *p*>0.050 and *F*_3,11_=1.27, *p*>0.050, respectively), and may therefore not have caused the observed differences.

**Figure 4:**
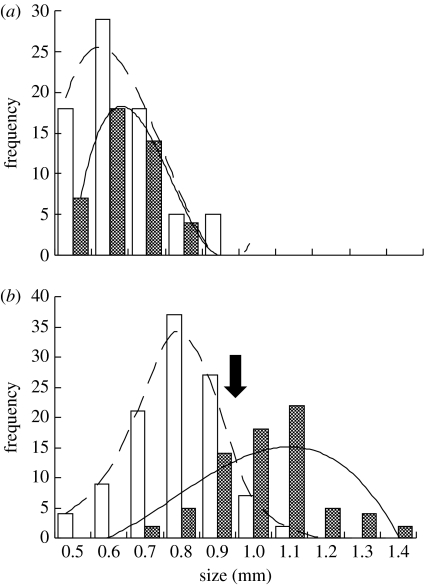
Size-dependent vertical distribution in natural systems. Frequency (number of individuals) of a small species (*a*) *D. cucculata* and a larger species. (*b*) *Daphnia galeata* in size classes from 0.5 to larger than 1.4 mm in surface and bottom waters of Lake Esch-Sur-Sure, Luxembourg (Thys & Hoffman 2005). Curves are fitted to data for clarity and arrow indicates where frequency curves cross, i.e. at which size the majority of *Daphnia* chose to avoid surface waters.

Size selective predation has since long been known to alter size distribution of zooplankton to a dominance of smaller species (Brooks & Dodson 1965). In this perspective, it is indeed intriguing that *Daphnia* individuals are able to make different risk assessments based on their own size, i.e. stay in deeper, darker waters as a refuge from predation risk at a size larger than approximately 0.9 mm and choose to feed in surface waters at smaller sizes. Hence, the *Daphnia* in our study were able to make size-specific adjustments regarding how to respond to a potential predator threat, and these experimental observations have support from natural systems and recalculations of previously published data suggest that this is a general phenomenon in nature. Hence, the depth distribution of one small (less than 1.0 mm) *Daphnia* species (*Daphnia cucculata*) and one larger species (*Daphnia galeata*; up to approx. 1.5 mm) was studied in Lake Esch-Sur-Sure, Luxembourg (Thys & Hoffman 2005). The small *Daphnia* species in their study showed an even size distribution between depths, whereas the larger species showed a strong difference in size class distribution between depths (figure 4). Hence, in accordance with our experimental results, recalculation of their data shows that small individuals preferred surface waters during the day, whereas larger individuals were almost exclusively found in bottom waters (Thys & Hoffman 2005). Interestingly, if the size-class distributions for the larger species are fitted to equations as was done for our data, the curves for surface and bottom waters cross at similar *Daphnia* size (approx. 0.9 mm) as in our study (see arrow; figure 2). Hence, in both experimental systems and in natural lakes, a majority of *Daphnia* individuals larger than approximately 0.9 mm make a risk assessment and behaviourally respond to predator risk by migrating downwards during daytime.

The results from our study may explain contradicting results regarding causes of variation in vertical distribution, e.g. DVM in zooplankton, sometimes showing strong diurnal migrations, sometimes no or only weak responses. That large, but not small, size classes of *Daphnia* migrate in response to predator cues may explain why studies not separating size classes may find no, or only weak, migratory responses to predation. Similarly, the very fine tuned response to UV threats, illustrated by figure 1, suggests that even minor and short-term alterations in the UV threat, such as diel alterations in algal biomass and depth distribution, or even cloudiness, may reduce or interrupt migratory behaviour. Hence, it may be of utmost importance to include size distribution of zooplankton when focusing on effects from predators, as well as assessing the instantaneous UV threat during sampling.

In conclusion, we show that UV radiation caused all size classes of *Daphnia* to avoid surface waters. Moreover, predation risk forced size classes of *Daphnia* larger than approximately 0.9 mm to reside at deeper darker waters, whereas smaller size classes continued to exclusively feed in surface waters during day. Our study shows that such simple organisms as invertebrate zooplankton are able to make individual, size-specific adjustments regarding how to respond to threats from both predators and UV radiation, which may be a way to maximize fitness (Lampert *et al*. 2003). Hence, we show that response compromises between the two lethal threats, predation and UV radiation, shape migratory patterns among zooplankton, insights that may considerably advance our mechanistic understanding of animal mass-migrations.

## Figures and Tables

**Table 1:** Vertical size distribution in response to predation risk. (Mean size (mm;±1s.d.) of *Daphnia* in surface (S) and bottom (B) waters of enclosures during the summer season. MANOVA is used to test differences in *Daphnia* size among treatments with (VF and UVF) and without (V and UV) predatory fish in surface and bottom waters, respectively.)

		no predation		predation		
				
depth		V	UV	VF	UVF	
June	S	0.81(0.28)	0.67(0.11)	0.75(0.25)	0.86(0.31)	*F*_1,12_=0.0; n.s.
	B	0.93(0.41)	0.87(0.25)	0.92(0.24)	0.86(0.31)	*F*_1,12_=0.14; n.s.
July	S	1.02(0.31)	0.99(0.27)	0.74(0.20)	0.73(0.07)	*F*_1,12_=17.7; *p*<0.001
	B	1.06(0.33)	1.34(0.38)	1.30(0.36)	1.20(0.28)	*F*_1,12_=0.42; n.s.
August	S	1.35(0.20)	1.26(0.35)	0.80(0.24)	0.75(0.15)	*F*_1,12_=23.88; *p*<0.001
	B	1.26(0.23)	1.47(0.19)	1.18(0.37)	1.28(0.26)	*F*_1,12_=2.99; n.s.
September	S	1.19(0.34)	1.16(0.19)	0.92(0.28)	0.79(0.20)	*F*_1,12_=9.77; *p*<0.009
	B	1.18(0.19)	1.25(0.32)	1.17(0.30)	1.14(0.36)	*F*_1,12_=0.17; n.s.
